# Perioperative Management of Cold Agglutinin Disease Using Central–Peripheral Temperature Monitoring: A Case Report

**DOI:** 10.1155/cria/4064832

**Published:** 2026-11-10

**Authors:** Cátia Sofia Silva, Ângela Zélia Oliveira, Claúdia Antunes, Susana Santos Rodrigues, Sara Marinho

**Affiliations:** ^1^ Department of Anaesthesiology Unidade Local de Saude Do Alto Ave Guimaraes Portugal

## Abstract

Cold agglutinin disease is characterised by temperature‐dependent immunoglobulin M–mediated complement activation and haemolysis. In severe disease, antibody activity may occur near physiologic temperatures. General anaesthesia impairs thermoregulation and promotes redistribution hypothermia, potentially exposing peripheral blood to temperatures associated with haemolysis. We describe the perioperative management of a 67‐year‐old man with high‐titre cold agglutinin disease undergoing right thyroid lobectomy. Because of baseline anaemia and anticipated transfusion constraints, a structured thermal management strategy was implemented. This included active prewarming before arrival in the operating room, ambient temperature control at 23°C, warmed intravenous fluids and continuous central and peripheral temperature monitoring. Core temperature remained between 37.2°C and 38.1°C throughout the procedure, while the central–peripheral temperature gradient was maintained at 0.4°C or less. No intraoperative hypothermia occurred, and haemodynamic stability was preserved without vasopressor support. Postoperative haemoglobin concentration remained unchanged at 8.8 g·dL^−1^, with no clinical evidence of haemolysis. This case suggests that perioperative management in cold agglutinin disease should focus not only on preventing overt hypothermia but also on limiting redistribution‐induced peripheral cooling. The combination of prewarming, environmental temperature control and dual‐site temperature monitoring was associated with thermal stability and an uncomplicated perioperative course.

## 1. Introduction

Cold agglutinin disease is a rare autoimmune haemolytic anaemia mediated by temperature‐dependent immunoglobulin M autoantibodies that bind erythrocyte surface antigens at reduced temperatures, leading to agglutination and complement‐mediated haemolysis [1, 2]. Disease severity is determined largely by antibody thermal amplitude, defined as the highest temperature at which antibody activity persists. In patients with broad thermal amplitude, reactivity may extend toward physiologic temperature ranges, rendering relatively small perioperative thermal fluctuations clinically relevant [1].

General anaesthesia profoundly disrupts thermoregulatory control. Following induction, peripheral vasodilation and inhibition of hypothalamic thermoregulation promote redistribution of core heat to peripheral tissues, producing an early decline in body temperature commonly termed redistribution hypothermia [3, 4]. Although this phenomenon typically occurs during the first hour of anaesthesia, its implications in cold agglutinin disease may extend beyond conventional concerns regarding systemic hypothermia. Peripheral cooling during redistribution may transiently expose circulating blood to temperatures capable of triggering cold agglutinin activation and complement‐mediated haemolysis, even when core temperature appears preserved.

In cardiac surgery, management strategies for patients with cold agglutinins are well established because cardiopulmonary bypass permits controlled systemic temperature regulation and active intravascular rewarming [5]. By contrast, perioperative temperature management during noncardiac surgery depends entirely on external warming measures and anaesthetic technique, and evidence guiding strategies specifically aimed at limiting redistribution‐induced peripheral cooling remains scarce.

We describe the anaesthetic management of a patient with high‐titre, broad thermal amplitude cold agglutinin disease undergoing right thyroid lobectomy. A structured multimodal thermal management strategy incorporating active prewarming, environmental temperature control, warmed fluids and continuous central–peripheral temperature monitoring was implemented to maintain thermal stability throughout the perioperative period.

## 2. Case Presentation

Written informed consent for publication was obtained from the patient.

A 67‐year‐old man was scheduled for elective right thyroid lobectomy with isthmectomy due to progressive compressive symptoms secondary to structural thyroid disease.

The patient had been diagnosed with cold agglutinin autoimmune haemolytic anaemia. Haematologic evaluation at the time demonstrated positive direct and indirect antiglobulin tests, panagglutination, high antibody titres and broad thermal amplitude consistent with clinically significant cold agglutinin disease. Given baseline anaemia and anticipated crossmatch complexity, the haematology team recommended reserving red blood cell transfusion for exceptional circumstances.

Relevant comorbidities included Type 2 diabetes mellitus, arterial hypertension, dyslipidaemia, obesity (body mass index of 30 kg m^−2^), severe obstructive sleep apnoea treated with continuous positive airway pressure and chronic corticosteroid therapy with prednisolone 20 mg daily. Preoperative haemoglobin concentration was 8.7 g·dL^−1^. Transthoracic echocardiography demonstrated a mildly reduced left ventricular systolic function with an ejection fraction of 46%. The patient was classified as American Society of Anesthesiologists Physical Status III.

Given the risk of haemolysis associated with peripheral cooling, perioperative management focused on limiting redistribution hypothermia and maintaining a thermal safety margin above the presumed antibody activation range.

Preoperative planning involved coordination among the surgical, anaesthesia and transfusion medicine teams. The blood bank was notified in advance, and compatible blood products were prepared with warming capability available if required. The procedure was scheduled as the first case of the day, and a multimodal thermal management strategy was implemented. This included active prewarming for 30 min before arrival in the operating room, transport on a prewarmed stretcher, ambient temperature maintained between 22°C and 24°C, warmed intravenous and irrigation fluids and continuous dual‐site temperature monitoring.

Standard monitoring was applied together with continuous central and peripheral temperature monitoring using an oesophageal temperature probe and a skin thermistor placed at the temporal region. Baseline core temperature was 37.4°C. An arterial catheter was not inserted because a short‐duration procedure with limited expected blood loss and stable haemodynamic conditions was anticipated. Continuous noninvasive monitoring was considered sufficient, and serial blood sampling was not expected to alter intraoperative management. General anaesthesia was induced with fentanyl, propofol and rocuronium and maintained with sevoflurane in an oxygen–air mixture. Hydrocortisone was administered intraoperatively in view of the patient’s chronic corticosteroid therapy. Mechanical ventilation was performed using pressure‐controlled ventilation with volume guarantee. Ventilatory parameters were adjusted throughout the procedure to maintain normocapnia and normoxaemia, guided by end‐tidal carbon dioxide monitoring and pulse oximetry.

Prewarming was applied for 30 min using forced‐air warming set to 43°C. Intraoperatively, active warming was maintained at the same setting, and intravenous fluids were administered via a fluid warmer set to 37°C. Following induction, the lowest recorded core temperature was 37.2°C. During the 87‐min procedure, central temperature remained between 37.2°C and 38.1°C, while peripheral temperature ranged from 37.4°C to 37.7°C (Figure 1). The central–peripheral temperature gradient (ΔT) remained ≤ 0.4°C throughout the procedure.

**FIGURE 1:**
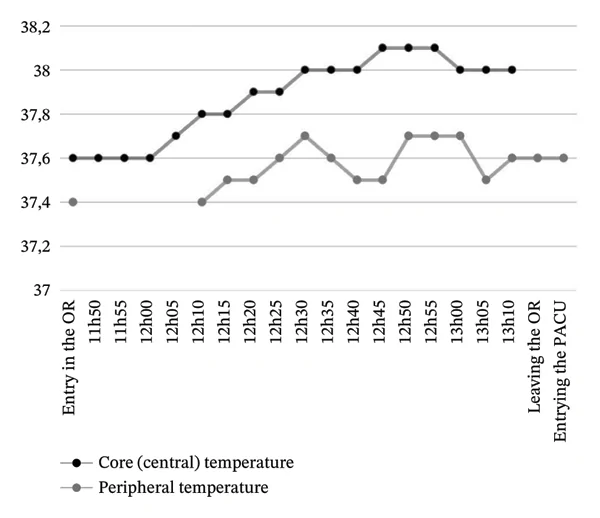
Continuous intraoperative central and peripheral temperature monitoring demonstrating maintenance of normothermia and minimal core‐peripheral gradient, suggesting effective attenuation of redistribution hypothermia. OR, operating room; PACU, postanaesthesia care unit.

Haemodynamic stability was maintained without vasopressor support. No additional blood‐sparing measures were used, with blood conservation relying on meticulous surgical haemostasis. Estimated blood loss was 150 mL, and transfusion was not required. Recovery and extubation were uneventful. At operating room exit, central temperature was 38.0°C, and peripheral temperature was 37.6°C.

Upon arrival in the postanaesthesia care unit, peripheral temperature was 37.6°C. Active warming was continued to avoid delayed hypothermia, and temperature at discharge from recovery was 37.2°C.

On postoperative day one, haemoglobin concentration remained stable at 8.8 g·dL^−1^, bilirubin levels were unchanged, and no clinical evidence of haemolysis was observed. The postoperative course was uneventful, with no signs suggestive of acute haemolytic exacerbation.

## 3. Discussion

Cold agglutinin disease presents a distinctive perioperative challenge because haemolytic risk depends not solely on preservation of systemic normothermia but on whether peripheral blood temperatures transiently enter the thermal range of antibody activity. Cold agglutinins are predominantly IgM autoantibodies, most commonly directed against the I antigen on erythrocytes. Their clinical significance is determined largely by thermal amplitude, defined as the highest temperature at which the antibody remains reactive with red cells. Although cold agglutinins typically react most strongly at low temperatures, a thermal amplitude above approximately 28°C–30°C is generally considered clinically significant because antibody binding may then occur within the cooler peripheral circulation. In some patients, reactivity may extend toward physiological temperatures [1, 2]. Following IgM binding, the classical complement pathway is activated, with C3b deposition on the erythrocyte surface. IgM subsequently dissociates as the blood returns to warmer central circulation, whereas complement remains bound and may promote predominantly extravascular haemolysis [1, 2]. Consequently, prevention of overt hypothermia alone may be insufficient to mitigate haemolytic risk during general anaesthesia.

In our patient, previous haematological assessment had documented high antibody titres and broad thermal amplitude. However, a specific thermal amplitude value was not available at the time of surgery. Temperature targets were therefore selected conservatively rather than according to a patient‐specific thermal threshold.

Perioperative haemolysis may be particularly consequential in cold agglutinin disease, as transfusion support is frequently complex and delayed by serologic incompatibility, especially in patients with baseline anaemia.

Anaesthetic‐induced vasodilation promotes redistribution of heat from the core to peripheral tissues, producing early redistribution hypothermia [3, 4]. Although typically regarded as a transient and clinically modest consequence of general anaesthesia, redistribution physiology may acquire particular relevance in cold agglutinin disease because antibody‐mediated agglutination occurs predominantly within cooler peripheral circulation. Consequently, peripheral blood may transiently enter the thermal range of antibody activity despite preserved core normothermia [1, 2]. This physiologic dissociation provided the rationale for continuous dual‐site temperature monitoring, since isolated core temperature assessment may inadequately reflect thermal conditions within peripheral compartments during redistribution phases [3, 4].

From this perspective, perioperative thermal management in cold agglutinin disease may be conceptualised less as maintenance of absolute normothermia and more as limitation of redistribution‐related thermal disequilibrium between central and peripheral compartments. In the present case, multimodal thermal management incorporating prewarming, environmental temperature control, warmed fluids and continuous dual‐site monitoring was associated with maintenance of a consistently narrow central–peripheral temperature gradient (≤ 0.4°C in this case) throughout surgery. Among warming interventions, prewarming may be particularly important. Sessler et al. demonstrated that approximately 30 min of forced‐air preinduction warming produces a clinically significant increase in peripheral tissue heat content, thereby attenuating redistribution‐related heat transfer following anaesthetic‐induced vasodilation [3, 6]. In cold agglutinin disease, this effect may be especially relevant, as attenuating peripheral cooling directly reduces the extent to which peripheral blood transiently enters the antibody’s thermal activation range.

Most published perioperative experience in cold agglutinin disease derives from cardiac surgery, where cardiopulmonary bypass permits direct systemic temperature manipulation and rapid thermal correction [5, 7]. In contrast, noncardiac surgery offers no equivalent mechanism for rapid thermal correction once redistribution has occurred, making anticipatory external warming strategies particularly important. Under these circumstances, perioperative temperature management depends primarily on prevention of peripheral cooling through prewarming, environmental temperature control and warmed fluid administration. Detailed descriptions of redistribution‐targeted thermal strategies in noncardiac surgery remain limited, and simultaneous central and peripheral temperature monitoring is infrequently reported. The present case therefore contributes quantitative perioperative temperature data supporting the feasibility of maintaining near‐isothermal central and peripheral compartments during noncardiac surgery.

Preoperative coordination with the transfusion medicine service is a further cornerstone of management in cold agglutinin disease. Because cold‐reactive autoantibodies can interfere with routine compatibility testing, established practice is to perform antibody screening at 37°C and to keep blood specimens warmed (37°C–38°C) from collection until serum has been separated, in order to preserve testing sensitivity [8]. When transfusion is required, use of an in‐line blood warmer is recommended, and the infusion extremity should be kept warm [8]. In the present case, advance notification of the blood bank and preparation of compatible blood products reflected this coordinated approach, and a line warmer was available if required, even though transfusion ultimately proved unnecessary.

This report has some limitations. As noted above, a patient‐specific antibody thermal amplitude was not available at the time of surgery, and temperature targets were therefore selected conservatively rather than individually tailored. In addition, haemolytic markers, including lactate dehydrogenase and haptoglobin, were not systematically assessed postoperatively, limiting the ability to exclude subclinical haemolysis. Nevertheless, the absence of clinically apparent haemolysis and the maintenance of stable central and peripheral temperature profiles support the feasibility of this perioperative management approach.

In summary, this case supports a perioperative approach to cold agglutinin disease focused on limiting redistribution‐induced peripheral cooling rather than solely preventing overt hypothermia. From a physiologic perspective, the objective may not be maintenance of normothermia alone but limitation of redistribution‐related peripheral thermal exposure during anaesthesia. In the present case, multimodal thermal management incorporating prewarming, environmental control, warmed fluids and continuous dual‐site temperature monitoring was associated with maintenance of thermal stability and absence of clinically apparent haemolysis during noncardiac surgery. Further studies incorporating systematic haemolysis biomarkers and distal peripheral temperature assessment are required to determine whether compartmental thermal management may reduce perioperative haemolytic risk in this population.

## Funding

No external funding was received for the preparation of this manuscript.

## Consent

Written informed consent for publication of this case report was obtained from the patient.

## Conflicts of Interest

The authors declare no conflicts of interest.

## Supporting Information

Additional supporting information can be found online in the Supporting Information section.

## Supporting information


**Supporting Information** CARE checklist (filled out).

## Data Availability

Data sharing is not applicable to this article as no datasets were generated or analysed during the current study.
